# Causes, consequences and clinical significance of aneuploidy across melanoma subtypes

**DOI:** 10.3389/fonc.2022.988691

**Published:** 2022-10-06

**Authors:** Eva R. Shteinman, James S. Wilmott, Ines Pires da Silva, Georgina V. Long, Richard A. Scolyer, Ismael A. Vergara

**Affiliations:** ^1^ Melanoma Institute Australia, The University of Sydney, Sydney, NSW, Australia; ^2^ Faculty of Medicine and Health, The University of Sydney, Sydney, NSW, Australia; ^3^ Charles Perkins Centre, The University of Sydney, Sydney, NSW, Australia; ^4^ Cancer & Hematology Centre, Blacktown Hospital, Blacktown, NSW, Australia; ^5^ Department of Medical Oncology, Royal North Shore and Mater Hospitals, Sydney, NSW, Australia; ^6^ Tissue Pathology and Diagnostic Oncology, Royal Prince Alfred Hospital and New South Wales (NSW) Health Pathology, Sydney, NSW, Australia

**Keywords:** melanoma, aneuploidy, chromosome missegregation, driver mutations, diagnosis, prognosis

## Abstract

Aneuploidy, the state of the cell in which the number of whole chromosomes or chromosome arms becomes imbalanced, has been recognized as playing a pivotal role in tumor evolution for over 100 years. In melanoma, the extent of aneuploidy, as well as the chromosomal regions that are affected differ across subtypes, indicative of distinct drivers of disease. Multiple studies have suggested a role for aneuploidy in diagnosis and prognosis of melanomas, as well as in the context of immunotherapy response. A number of key constituents of the cell cycle have been implicated in aneuploidy acquisition in melanoma, including several driver mutations. Here, we review the state of the art on aneuploidy in different melanoma subtypes, discuss the potential drivers, mechanisms underlying aneuploidy acquisition as well as its value in patient diagnosis, prognosis and response to immunotherapy treatment.

## Introduction

**Figure 1:**
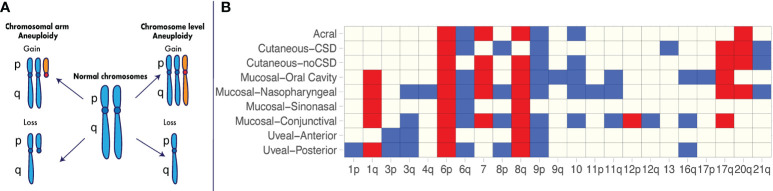
Recurrent aneuploidy events in melanoma. **(A)** Chromosome and chromosomal arm aneuploidy. Gained arms and chromosomes are illustrated in orange. Lost arms and chromosomes are depicted by a reduction in chromosomal content with respect to a normal karyotype. **(B)** Recurrent aneuploidy events across melanoma subtypes. Chromosomal arm and whole-chromosome gains and losses commonly observed within a specific melanoma subtype are indicated in blue and red, respectively. Recurrent events are illustrated as identified across studies in acral melanomas (3, 4), cutaneous melanomas with and without chronic sun damage (CSD) (3), mucosal melanomas including nasopharyngeal (3), conjunctival (5, 6), sinonasal (7, 8) and oral cavity melanomas (9), uveal posterior (10) and uveal anterior melanomas (11–14).

In the final stage of the cell cycle, the genomic content of a cell must be accurately duplicated, segregated, and then partitioned into two physically distinct daughter cells. Errors in this process can lead to aneuploidy, a state of the cell in which the number of either whole chromosomes or chromosome arms becomes imbalanced (1), resulting in a deviation of the DNA content from the euploid state (2) (**Figure 1A**).

Aneuploidy was first observed in the late 19^th^ century by David von Hansemann (15), who noted the presence of asymmetrical nuclear divisions in epithelial tumor cells with chromosomes that had failed to fully segregate (16). Building on these observations, Theodor Boveri conducted a series of experiments on sea urchin eggs, and established a causal link between chromosomal aberrations and the development of malignant tumors (17). Since then, and as technologies have enabled a higher resolution of the extent of alterations in the cancer genome, an aneuploid state, as well as varying levels of aneuploidy, have been identified in the majority of cancers (18). Across melanoma subtypes, varying levels of aneuploidy are observed with common and specific recurring aneuploidy events identified (3), indicative of an underlying ubiquitous and tumor type-specific driver role in disease progression.

The association between an aneuploid state and poor prognosis has been described in melanoma and other cancer types in treatment-naïve and treatment-exposed settings (19–22). An aneuploid state has been found more frequently in cutaneous melanomas compared to benign melanocytic tumors (23), and has been associated with important pathological factors as well as disease recurrence and survival (20). In uveal melanoma, the presence of specific chromosomal gains and losses identifies clinically-relevant groups regarding risk of metastasis and survival (24, 25). In the context of immune checkpoint inhibitor (ICI) therapies, aneuploidy has been reported to be predictive of response to treatment (22).

Several defects in mechanisms of the cell cycle that can result in chromosomal mis-segregation and aneuploidy have been identified in melanoma. Importantly, key driver mutations such as *BRAF* V600E (26, 27), *KIT* K642E (28) and loss of *CDKN2* (29) have been implicated with such defects including dysregulation in metaphase to anaphase transition, replication stress and centrosomal amplification.

In this review, we provide an overview of the studies quantifying aneuploidy across melanoma subtypes, discuss its diagnostic, prognostic and predictive value as well as the mechanistic defects that result in aneuploidy.

## Reports of aneuploidy in melanoma

Historically, the resolution with which aneuploidy can be observed has been a function of the technologies available. Early studies on small cohorts relied on karyotyping techniques such as G-banding as well as flow cytometry. Later studies using fluorescent *in situ* hybridization (FISH) (30, 31), single nucleotide polymorphism (SNP) arrays (32–34), comparative genomic hybridization (CGH) (3, 35–37) and next-generation sequencing technologies (4, 38–42) on large cohorts have provided an unprecedented resolution to evaluate the extent of aneuploidy in melanoma and other cancers.

Early reports of aneuploidy in melanoma were based on cytogenetics techniques such as G-banding and Q-banding, which allow for the visualization of the karyotype. In one of the earliest studies utilizing these techniques in a metastatic melanoma tumor, Chen & Shaw noted only one identifiable copy of chromosomes 1, 2, 8, 9, 10, 11, and 13 (43). Subsequent studies consistently documented the presence of aneuploidy in melanoma. In a study of 17 lesions spanning five nevi, two primary and 10 metastatic melanomas, Balaban et al. (44) noted normal karyotypes in benign melanocytic lesions and frequent alterations of chromosomes 1p, 6, and 7 in melanomas. In a larger cohort of 37 patients, the researchers confirmed these findings (45) and noted common as well as additional aneuploidy events and other chromosomal abnormalities in metastases compared to the matched primary tumors in four patients (45). More recently, Shain et al. demonstrated a significant increase in the extent of aneuploidy (as measured by the fraction of the genome affected by copy number alterations) during the transition to invasive disease utilizing a cohort of matched primary melanomas, their adjacent precursors as well as matched primary and regional metastases (46). A sequential order of distinct aneuploidy events occurring at different stages of melanoma progression was observed in this study, with specific alterations such as gain of chromosome 6p appearing particularly early. Recent work in matched primary and metastasis melanomas using next generation sequencing technologies has indeed confirmed that large-scale aneuploidy, including loss of heterozygosity (LOH) – the irreversible loss of an allele - are prominent features of late-stage metastatic cutaneous melanoma, increasing in extent from early stage to advanced melanoma as it progresses in many patients (42).

Further studies utilizing newer technologies in large cohorts have enabled identification of recurrent aneuploidy events in specific melanoma subtypes (**Figure 1B**). In one of the earliest major studies of its kind using array CGH (3), 126 primary melanomas were characterized across a range of different subtypes, including acral melanomas – those originating on the palms, soles and nail bed – as well as mucosal melanomas – those occurring within the mucous membranes. The cohort consisted of acral melanomas (n = 36), mucosal melanomas [mainly nasopharyngeal (n = 20)], cutaneous melanomas from chronic sun-induced damaged (CSD) areas (n = 30), and cutaneous melanomas from non-sun-induced damaged (non-CSD) areas (n = 40). The authors found major differences in recurrent specific aneuploidy events between CSD and non-CSD cutaneous melanomas, including losses on chromosome 10 and gains of chromosome 7 and 8q in the latter group. The acral and mucosal melanoma subtypes showed the greatest extent of aneuploidy, as measured by the overall proportion of the genome affected by DNA gain and losses (3). However, acral and mucosal melanomas differed in the chromosomal regions that were affected (**Figure 1B**). For example, mucosal lesions presented with significantly more gains on chromosomes 1q, and 17q, and losses on 3q, 8p, and 11p (**Figure 1B**) compared to acral lesions (3). Even within these non-cutaneous subtypes, there are differences in the extent of aneuploidy and the specific recurring events observed. In a recent study by Newell et al. using whole-genome sequencing on 87 acral melanoma lesions (4), a high extent of aneuploidy was confirmed to be present in this subtype, occurring at a greater level in subungual – those involving the nail bed - acral melanomas compared to non-subungual acral melanomas (4). In mucosal melanomas, the recurring events identified across studies vary depending on the anatomical location (**Figure 1B**).

These and additional studies in the last two decades (4–14, 41) have made evident the heterogeneous aneuploidy landscape across cutaneous and non-cutaneous melanomas as well as the extent to which recurring aneuploidy events are ubiquitous across subtypes or specific to melanoma subtypes. Some recurrent aneuploidy events such as gain of 6p, gain of 8q and loss of 9p are nearly ubiquitous across melanoma subtypes and may confer increased tumor ‘fitness’ independently of anatomical site. Others are mostly recurrent within subsets (e.g., gain of 1q and loss of 11q within mucosal subtypes) or within specific subtypes (e.g., loss of chromosome 13 within CSD cutaneous melanomas) and may be selected to drive disease in a site-specific or tissue-specific manner.

Several studies have explored the potential drivers of recurring aneuploidy events in cutaneous and non-cutaneous melanoma. Rakosy et al. (47) utilized a cohort of 36 primary cutaneous melanomas to identify 1,080 genes differentially expressed between ulcerated and non-ulcerated melanomas. Ulceration is an important pathological factor used for staging of primary melanoma, defined as the full thickness absence of an intact epidermis above any portion of the primary tumor with an associated host reaction (48). Assessment of the association between copy number alterations and gene expression in 17 melanomas identified 150 genes whose expression correlated with copy number losses in ulcerated tumors, with an enrichment of genes downregulated in chromosomal arms 6q and 10q. Several of these genes are implicated in cell-cell and cell-matrix adhesion, as well as apoptosis. Kwong and Chin (49) identified genes downregulated in metastatic melanomas compared to primary cutaneous melanomas and nevi whose downregulation was associated with copy number loss. *In vivo* and *in vitro* RNA interference experiments demonstrated tumor-suppressive capabilities for growth and invasion for several genes in chromosome 10, suggesting that loss in this chromosome may target multiple tumor suppressors in melanoma. Along these lines, a recent study using whole-exome and whole-genome sequencing of matched early-stage and advanced stage cutaneous melanomas (42) identified large genomic regions undergoing LOH through disease progression, resulting in a reduction of the neoantigenic load. In many cases, the LOH event resulted in the simultaneous loss of several putative neoantigenic mutations. Together, these studies suggest that chromosomal losses may be an efficient mechanism to modulate the dosage of genomic features that contribute to melanoma pathogenesis and disease progression.

In posterior uveal ocular melanoma, loss of chromosome 3 and gain of 8q are associated with expression profiles that define a patient group with worse survival, named *class 2* (24, 25). The strong correlation between monosomy of chromosome 3 and *BAP1* mutations (50) indicates inactivation of this tumor suppressor is a driver of this aneuploidy event. Other studies have implicated gain of 8q with increased expression of the *MYC* oncogene (51) and *PTK2* (52), a gene that encodes the focal adhesion kinase (FAK), shown to mediate Gαq-driven YAP activation in uveal melanomas. In contrast, prior studies (53, 54) focused on HLA expression have reported no associations between 6p status – commonly amplified in *class 1* uveal melanomas - and expression of members of the Major Histocompatibility Complex (MHC), where it resides. Overall, further studies within cutaneous and non-cutaneous melanoma subtypes are needed to understand the drivers underlying the recurring aneuploidy events observed.

## Diagnostic, prognostic and predictive value of aneuploidy

**Figure 2:**
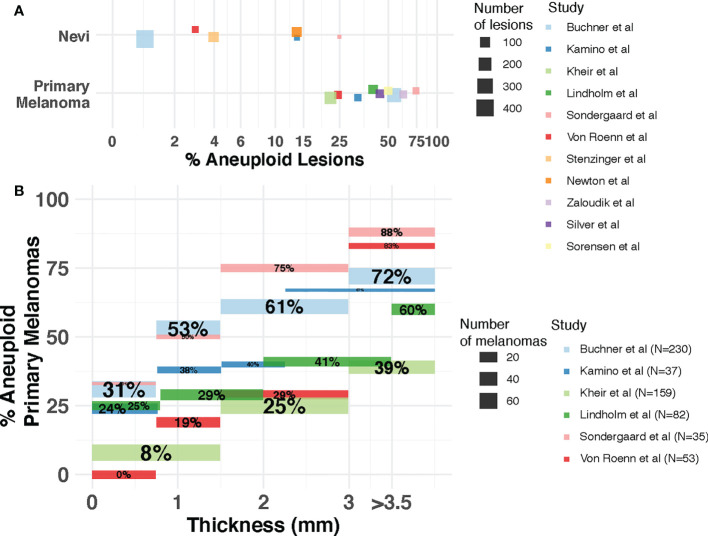
Incidence of aneuploidy in cutaneous nevi and melanoma. **(A)** Aneuploidy in nevi and primary melanomas reported by flow cytometry studies. Each dot represents a study. The size of the dot is proportional to the number of lesions (nevi or melanoma) in that study. Position of dots has been jittered vertically for better visualization. **(B)** Incidence of aneuploidy across thickness. The percentage of aneuploid melanomas reported (y-axis) by each study for each thickness level (x-axis) is represented as a segment. Segment size is proportional to the number of patients in each thickness level for each study. Percentages withing segments indicate the proportion of patients within each thickness level that are aneuploid. Studies included are Kamino et al. (60), Lindholm et al. (61), Buchner et al. (23), Kheir et al. (20), Sondegaard et al. (55), Von Roenn et al. (57), Stenzinger et al. (56), Newton et al. (58), Sorensen et al. (63), Zaloudik et al. (59) and Silver et al. (62).

The question of whether the presence and extent of aneuploidy is associated with disease progression, and whether it could inform patient diagnosis, prognosis and response to treatment, has been explored in cutaneous melanomas. Early studies using flow cytometry (20, 23, 55–63) revealed the incidence of aneuploidy was much lower in benign melanocytic lesions compared to primary melanomas (**Figure 2A**), opening a potential avenue for this DNA property to be used for diagnostic purposes to elucidate malignancy of a lesion. Across these studies, 1-25% of the nevi lesions were aneuploid. In contrast, the percentage of aneuploid lesions ranges from 17%-75% in primary melanomas. Importantly, in two of these studies the aneuploid melanocytic nevi (1/34 and 4/16 by van Roenn et al. and by Sordengaard et al., respectively) were regarded as “undoubtedly benign” (55, 57). In two other studies (23, 56) the aneuploid melanocytic nevi identified (4/422 by Buchner et al., 4/101 by Stenzinger et al.) were congenital nevi as opposed to acquired nevi (46 and 39 total congenital nevi assessed by Buchner et al. and Stenzinger et al., respectively). In the study by Newton et al. (58), large variability in the extent of aneuploidy among nevi types was observed, with 7% (3/44) of melanocytic cellular nevi, 30% (6/20) of dysplastic nevi, 11% (1/9) of small congenital nevi and 50% (4/8) of giant congenital nevi being aneuploid. These findings suggest that aneuploidy alone may be insufficient as a property of the cells to identify malignancy in borderline melanocytic lesions (57), and that cellular heterogeneity needs to be taken into account.

Aneuploidy has also been associated with pathological factors indicative of disease progression in primary melanomas (**Figure 2**). Thickness - measured in millimeters from the epidermal granular layer to the deepest point of invasion – is the most important prognostic factor for primary cutaneous disease (64) and it is the main pathological factor that defines the T (Tumor) category of the American Joint Committee on Cancer (AJCC) staging system (48). Buchner et al. (23) utilized flow cytometry on 230 primary melanomas, identifying a higher incidence of aneuploidy associated with increased tumor thickness with an incidence of 31.3% in thin tumors (<0.75 mm.) and 72.4% in thick tumors (>3 mm.). Several other studies using flow cytometry (20, 55, 57, 60, 61) confirmed this association between aneuploidy and thickness using cohorts of varying size (**Figure 2B**) and thickness distributions. One study using a large cohort (20) also reported a highly significant association with ulceration status, among other pathological features. As the presence of ulceration is associated with thicker melanomas (65), the underlying role of aneuploidy on the biology of distinct pathological factors of prognostic relevance deserves further study.

### Prognostic value of aneuploidy in melanoma

The prognostic value of aneuploidy has been assessed in primary cutaneous melanomas. The study by Kheir et al. (20) identified aneuploidy to be strongly associated with higher recurrence rate and shorter disease-free survival in a cohort of 159 primary cutaneous melanomas. Interestingly, this association was significant within the groups of thinner (<1.5 mm; 57 patients) and thicker (>2.9 mm; 33 patients) disease, but not in those patients with intermediate thickness (55 patients), albeit a non-statistically significant trend of higher recurrence rate in aneuploid melanoma patients within this group. Furthermore, ploidy status was identified as a significant factor in a multivariate model with other clinical-pathological factors. Other studies with smaller cohorts have shown disagreement regarding the prognostic value of aneuploidy. The study by Silver et al. (n=63) (62) identified ploidy status as a significant factor for recurrence in multivariable analyses with thickness and other variables. In contrast, the study by Lindholm et al. (n=82) (61) identified a significant association between aneuploidy and survival, but this association was not observed within thickness groups or in multivariable analyses with other clinical-pathological factors. The study by Zaloudik et al. (n=50) (59) did not identify aneuploidy as relevant for survival. These disagreements may be explained by differences in thickness representation across primary melanomas, sample size, follow-up of clinical progression as well as different survival endpoints.

The prognostic value of specific aneuploidy events has been identified in primary uveal ocular melanomas. Patients presenting with loss of chromosome 3 tend to have worse prognosis than those who do not (66) while gain of chromosome 6p is associated with better prognosis (67). Alterations in chromosome 8q have been associated with worse prognosis in some studies (67, 68), but not in others (66). In contrast, measuring overall levels of aneuploidy has not been found to be as informative as specific aneuploidy events for prognosis of uveal melanoma (66, 69).

Currently, there is limited information of the prognostic value of aneuploidy for patients with acral and mucosal melanoma subtypes. In a cohort of 46 subungual acral melanomas (70), a higher percentage of aneuploid cells was associated with worse survival in univariate analysis, but not in multivariate analysis. In a cohort of vulvar melanomas (n=43), aneuploid disease was associated with worse survival in univariate and multivariate analyses (71). In a study of 19 primary sinonasal melanomas (8), a significant association was found between higher rates of copy number alterations – a proxy for a higher extent of aneuploidy - and shorter metastasis-free survival, with no association identified for overall survival.

### Aneuploidy as a predictor of response to immunotherapy in melanoma

The extent of aneuploidy has also been associated with response to ICI treatment. In Davoli et al. (22) and in the more recent work by Anagnostou et al. (72), aneuploidy levels were found to be higher in melanoma patients that did not respond to ICIs. By analyzing survival data from two clinical trials of anti-CTLA-4 blockade in metastatic melanoma patients, Davoli et al. found that higher aneuploidy levels were an independent predictor of poorer overall survival in these patients in a model that included mutational load (22). The study by Anagnostou et al. consisted of a cohort of metastatic melanoma patients either receiving anti-PD1 blockade alone, or anti-CTLA4 and anti-PD1 blockade in combination (72). Non-responders presented a trend of higher levels of aneuploidy compared to responders (72). Similarly, aneuploidy was associated with worse progression-free survival and overall survival, and this trend was particularly pronounced in the combination treatment group (72). Additionally, in a recent study by Newell *et al*., whole genome sequencing was pursued on 77 baseline (pre-treatment) biopsies from melanoma patients with advanced cutaneous disease treated with anti-PD1 or combination anti-PD1/anti-CTLA4 therapy in order to examine genomic associations with response to ICI treatment (73). The authors found that overall, poor response to ICIs was significantly associated with increased structural variant burden, and there was a trend towards an association with higher extent of copy number alterations. No association was identified between response to ICIs and whole-genome doubling (WGD) (73), which is a known correlate of aneuploidy (18). It remains unknown whether aneuploidy levels or specific aneuploidy events may be linked to response to ICIs in acral, mucosal, and uveal melanoma subtypes. Studies focused on non-cutaneous melanoma subtypes are needed, as ICI therapies have produced lower response rates (74–76) than in cutaneous melanomas (77–79), with 45-62% objective response rates (ORR) reported in cutaneous disease versus 12-44%, 29-35% and 3.6-5% ORR in acral, mucosal and uveal melanomas, respectively. These results prompt the urgent need to identify patients that will fail ICI.

Further studies with sufficient statistical power to evaluate the diagnostic, prognostic and predictive value of aneuploidy and specific events across melanoma subtypes hold promise to better stratify patients, and ultimately impact their management.

## Mechanisms that lead to aneuploidy

**Figure 3:**
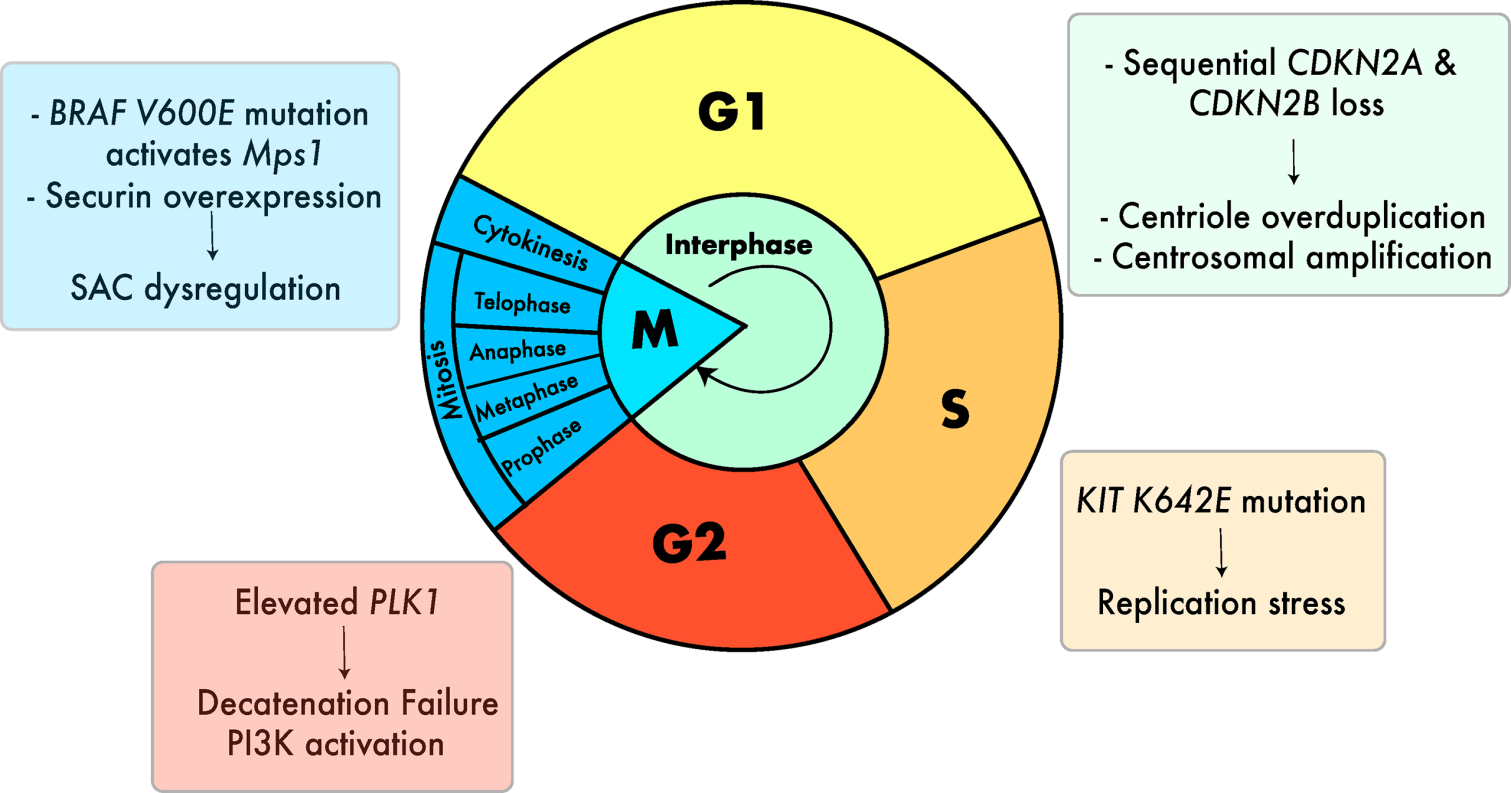
Cell cycle defects that can lead to aneuploidy in melanoma. Defects include centrosomal amplification (29, 81, 82), replication stress (28), G2 decatenation failure (83–85) and SAC dysregulation (26). (27, 86). Colors of the boxes containing text are associated with the color of the respective stage of the cell cycle.

Several cell-intrinsic checkpoints exist to ensure that the major events of the cell cycle take place without error. These checkpoints function as stress-response mechanisms by detecting errors and preventing the next stage of the cycle from taking place until the error is resolved (80). Defects in these mechanisms can lead to mitotic errors that result in incorrect chromosome segregation and chromosomal gains and losses in both the dividing cell and subsequent daughter cells (80). In melanoma, several defects in these mechanisms have been described that can lead to aneuploidy (**Figure 3**).

### Errors during metaphase to anaphase transition and driver mutations

The role of the mitotic checkpoint, or spindle assembly checkpoint, is to prevent the onset of anaphase until each of the sister chromatids is properly attached to the mitotic spindle (87). Mutations and impaired signaling of the mitotic checkpoint can lead to chromosomal mis-segregation and aneuploidy (87). Evidence for defective mitotic checkpoint signaling has been found in a range of different cancers, including colon cancer (88), breast cancer (89), and gastric cancer (90).

The protein securin (encoded by *hPTTG1)* an inhibitor of separase, is required for sister chromatid separation during mitosis. Studies have shown that overexpression of securin can prevent the separation of the sister chromatids, causing the daughter cell to become aneuploid (91). Its overexpression has also been reported to prevent the tumor-suppressor protein p53 from inducing cell death (92), which could allow aneuploid cells to evade destruction despite their genetic instability. The association between securin overexpression and aneuploidy is now well established (91, 93, 94), although melanoma-specific studies are limited. In one study, securin was found to be significantly overexpressed in primary cutaneous melanoma cells in comparison with nevi, and this overexpression was found to be particularly pronounced in the nodular melanoma subtype (86). Moreover, securin overexpression was correlated significantly with DNA content, with securin levels being particularly high in lesions presenting large, highly atypical melanoma cells with multiple nuclei (86).

In melanoma, the *BRAF* V600E mutation has been proposed as a source of deregulated mitotic checkpoint signaling. *BRAF* mutations are extremely common in cutaneous melanoma, with approximately 40% of patients harboring a *BRAF* mutation, most commonly the V600E genotype (39). In their 2008 study, Cui & Guadagno demonstrated that BRAF signaling regulates the activity of monopolar spindle 1 *(Mps1)* - an essential activator of the mitotic checkpoint – *via* the MAPK pathway (26). When the BRAF V600E mutation was introduced into wild-type BRAF melanoma cells, *Mps1*-associated kinase activity increased 10-fold (26). By sustaining high levels of *Mps1-*associated kinase activity, BRAF V600E prolonged activation of the mitotic checkpoint, delaying mitotic progression (26). The authors argued that this could lead to genomic instability and errors in chromosome segregation (26). In a later study by the same group, it was shown that introducing oncogenic mutant BRAF V600E into human melanoma cells led to high levels of aberrant spindles, supernumerary centrosomes, and the mis-segregation of chromosomes (27). When a MAPK inhibitor was introduced, these mitotic defects were suppressed, supporting the hypothesis that the mitotic effects of *BRAF* occur in a MAPK-dependent manner (27).

A recent study (28) utilizing a murine allograft model of the most common *KIT* missense mutation – K642E - showed that *KIT* mutation induces high levels of replication stress, leading to reduced replication fork speed and elevated DNA damage in S-phase. Across 674 samples from 11 melanoma studies, the authors identified genetic interactions between *KIT* alterations and mutations in cell cycle checkpoint genes *ATM*, *ATR* and *CHEK1*. In contrast, no interaction was identified between these cell cycle genes and *BRAF* or *NRAS* mutations, suggesting that perturbations in DNA damage signaling may be of importance specifically in *KIT*-mutated melanomas (28). Replication stress has been proposed to result in chromosomal mis-segregation *via* transient multipolar spindles caused by premature centriole disengagement in cancer cells (95). In agreement with this, *KIT*-mutant cells harbored widespread chromosomal mis-segregations compared to their parental counterpart (28). In contrast to *BRAF* driver mutations, *KIT* driver mutations are present to a higher extent in acral and mucosal melanomas (10.8% and 11.5% respectively) compared to cutaneous melanomas (9.7% in CSD and 5.1% in non-CSD cutaneous melanoma) (96).

These studies suggest a role for *BRAF* and *KIT* driver mutations as well as key players of the mitotic cycle on the dysregulation of mechanisms that lead to aneuploidy across melanoma subtypes, and therefore prompt the need for further scrutiny into treatment options in the context of aneuploid disease.

### G2 phase decatenation failure

Another cell-intrinsic mechanism is the G2 phase decatenation checkpoint, whose role is to delay the progression of the cell cycle until the chromosomes have been fully decatenated, or disentangled, by topoisomerase II (97). If mitosis continues with catenated chromosomes undetected, this can lead to aneuploidy and chromosomal rearrangements in the daughter cells (97). In a study assessing the G2 decatenation checkpoint function in melanoma cell lines, it was shown that ~68% of the cell lines had either a partly (40%) or severely (28%) defective checkpoint (83), with defects in decatenation resulting in failure to separate the sister chromatids and tangled chromosomes. Spoerri et al. (85) identified a cell cycle checkpoint defect that leads to unstable checkpoint arrest characterized by intact ATM-CHK2 activation and elevated *PLK1* expression. However, despite the accumulation of DNA damage that it may cause, defects in the decatenation checkpoint do not necessarily lower a cell’s viability, due to the availability of compensatory mechanisms that the cell can adopt (84). In one study, it was shown that melanoma cell lines with defective decatenation checkpoints have an increased reliance on the Phosphatidylinositol-3-kinase - PI3K pathway, which is an intracellular pathway involved in the regulation of cell survival that can generate anti-apoptotic signaling even in the face of genotoxic stress (84). In cell lines with defective decatenation checkpoints, an increase in anti-apoptotic signaling *via* PI3K prevented cell death despite the presence of catenated chromosomes. Accordingly, inhibition of PI3K pathway led to apoptosis. These results may point to one of the mechanisms by which unstable aneuploid melanoma cells evade destruction.

### Centrosomal amplification

Several errors during the cell cycle including centriole overduplication, cytokinesis failure and cell fusion can lead to centrosome amplification (CA), resulting in the formation of multipolar spindles that disrupt mitotic fidelity and lead to aneuploidy (98). CA has been proposed to be one of the major causes of aneuploidy in cancer (98). Using immunofluorescence to visualize 79 melanoma tissue microarrays, Denu et al. (81) reported that CA is prevalent in melanoma and arises mainly from centriole overduplication (as opposed to cytokinesis failure or other mechanisms of failed mitosis), albeit a lack of association of expression of Polo-like Kinase 4 - *PLK4*, a main driver of centriole duplication – with CA. A later study utilizing melanoma cell lines across different stages of melanoma progression implicated sequential loss of Cyclin Dependent Kinase Inhibitor genes *CDKN2A* and *CDKN2B* with CA (29). Based on a centrosome duplication assay, double negative cell lines for p16 and p15 (encoded by *CDKN2A* and *CDKN2B*, respectively) overduplicated centrosomes, suggesting that both proteins need to be absent for centrosome overduplication to occur. Evidence for centrosome accumulation (as opposed to duplication, occurring after failed cell division) was reported based on DNA content analysis. Of note, loss of 9p21 – where *CDKN2A/B* reside - is prevalent in melanoma and has recently been associated with primary resistance to ICI treatment (99). Finally, a recent study (82) utilizing a cohort of 75 primary uveal melanomas showed amplified centrosome numbers as well as an increase in centrosomes with enlarged pericentriolar matrix (PCM) relative to the surrounding normal tissue, with PCM phenotype significantly associated with monosomy of chromosome 3. Uveal melanomas with monosomy of chromosome 3 have increased aneuploidy compared to those with disomy of the same chromosome, in agreement with a scenario of increased centrosomal abnormalities in the former group (82). The authors argue that prior observations that melanomas with monosomy of chromosome 3 have increased aneuploidy compared to melanomas with disomy of this chromosome (66) is consistent with increased centrosomal abnormalities in the former melanoma group.

While the exact extent to which different defects lead to CA across melanoma subtypes remain to be elucidated, these studies implicate yet another driver mutation - loss of *CDKN2* – in aneuploid disease and implicate CA with high-risk uveal melanoma disease.

## Unresolved issues

Since Boveri’s early experiments with sea urchin eggs, it has been well-established that aneuploidy is an important characteristic of most cancers. While substantial progress has been made in characterizing aneuploidy across melanoma subtypes, many questions remain regarding the underlying drivers, its diagnostic, prognostic and predictive value as well as the mechanisms that cause aneuploidy and that could lead to the identification of novel therapeutic avenues.

### Drivers of aneuploidy

Several studies have identified candidate drivers underlying recurring aneuploidy events across melanoma subtypes *via* matched RNA expression and copy number alterations of melanoma lesions. Tumor suppressors and oncogenes with a demonstrated role in melanoma tumorigenesis and progression lay on regions that are recurrently aneuploid, including the oncogenes *BRAF* (7q34) and *MYC* (8q24) and the tumor suppressors *BAP1* (3p21), *CDKN2A* (9p21) and *PTEN* (10q23). The extent to which alterations of chromosomal arms and whole chromosomes that include these regions are selected solely due to the presence of these specific genes as opposed to multiple targeting of several oncogenic and tumor-suppressive features within the same genomic location is unclear. A few studies have provided evidence for the latter scenario based on the assessment of *PTEN* and other putative tumor suppressors in chromosome 10 (49, 100). Studies probing the genomic and expression changes across matched longitudinal melanoma lesions from the same patient provide an opportunity to directly identify acquisition of aneuploidy events and their impact on the expression of oncogenes and tumor suppressors, as well as other factors that may impact tumor progression. Cutaneous melanomas are one of the cancers with the highest mutational loads (101), and aneuploidy acquisition in specific chromosomes has been hypothesized to modulate a competitive growth advantage *via* amplification and deletion of alleles carrying mutations that are beneficial (e.g. oncogenic mutations) or detrimental (e.g. neoantigenic mutations), respectively, to tumor fitness (42). In agreement with this, pan-cancer studies have shown evidence of positive selection for chromosomal imbalances associated with oncogenic driver mutations – including *BRAF* - as well as negative selection for imbalances associated with mutations in haplo-essential driver genes such as the Splicing Factor 3b Subunit 1 - *SF3B1*, (102) a gene frequently mutated in uveal melanomas as well as vulvovaginal and anorectal mucosal melanomas (103). The work by Laughney and colleagues (104) utilized an *in-silico* approach to show that tumors evolve toward a near-triploid state that maximizes oncogenicity and minimizes tumor-suppressiveness, in agreement with the observed frequent triploid state of melanomas and other cancer types. Future studies that expand our understanding of the underlying biological drivers of specific chromosomal regions as well as more general genomic configurations that may explain the differences in the overall extent of aneuploidy observed across melanoma subtypes carry the potential to identify new therapeutic targets.

### Diagnostic and prognostic value of aneuploidy

While aneuploidy detection *via* FISH and CGH is used for diagnosis in routine practice (105), the role of aneuploidy as a prognostic tool remains to be elucidated. The differences in aneuploidy prevalence reported across nevi types suggests that this cellular heterogeneity needs to be taken into consideration when evaluating this genomic property to estimate the degree of malignancy of a given melanocytic tumor. Additional molecular features combined with aneuploidy may be informative for this purpose. Recent studies comparing matched precursor lesions and primary cutaneous melanomas (106–108) have shown that mutations in non-coding promoter regions of genes as well as specific coding mutations and copy number changes of oncogenes and tumor suppressors can further inform on the likelihood of malignancy for a given lesion.

The positive association between aneuploidy and thickness reported by multiple studies needs to be considered in order to understand whether aneuploidy carries independent prognostic value for recurrence and survival. While increased thickness is generally associated with recurrence and death, recent large-scale clinical studies have revealed that the relationship between thickness, recurrence and survival is far more complex than initially thought. While melanomas with a thickness <2mm have very favorable 5-year survival rates (48), this group contributes most deaths given its high prevalence (109). In contrast, a significant proportion of patients with thick melanomas (>4 mm.) have good outcome (48). Recurrence in patients with thin melanomas often occurs much later than in patients with thicker disease (110, 111). Within thin melanomas (<1mm.), a thickness of 0.8 mm. is a critical threshold above which patients are at increased risk of recurrence (110). Within thick melanomas, a recent study shows that the relationship between thickness and survival reverses beyond 15 mm. (112). Furthermore, estimating the risk of recurrence and survival from primary disease is becoming increasingly relevant in the context of ICI treatments in the adjuvant setting as clinical trials show effectiveness in patients with clinically high-risk primary localized cutaneous melanoma (113). These clinical studies prompt the need for future work that auscultates aneuploidy in primary melanomas across the entire spectrum of thickness levels, utilizing a cohort that confers sufficient statistical power for recurrence and survival events, and with sufficient follow-up to include accurate endpoint information for patients with slow-progressing disease such as thin melanomas. In non-cutaneous disease, while immense progress has been made regarding the role of aneuploidy in posterior uveal melanomas, a currently underexplored area is the prognostic value of aneuploidy in melanomas occurring at different body sites within the mucosal and acral subtypes. This is hampered by the low incidence of these melanoma subtypes compared to cutaneous disease, and as such multi-institutional efforts are required to generate cohorts that are informative.

### Predictive value of aneuploidy and association with immune microenvironment

The association between aneuploidy and ICI treatment response reported across melanoma cohorts and treatment modalities suggests that further scrutiny of the biology of aneuploid tumor-immune cell interaction can further inform on its predictive value and new therapeutic avenues for non-responders that present with aneuploid disease. The study by Jung et al. showed that global methylation loss was associated with worse prognosis following treatment with ICIs in lung patients as well as in a cohort of 40 melanoma patients who received ICI treatment in The Cancer Genome Atlas (TCGA) melanoma study (114). In the same study, global methylation loss was significantly correlated with a higher extent of chromosomal and arm-level somatic alterations in melanoma TCGA patients and other cancers. An association between global methylation loss with immune signatures independent of aneuploidy levels was identified, leading the authors to suggest that the immune evasion of aneuploid tumors is associated with genomic demethylation (114).

Several studies have reported mechanisms that enable the detection and elimination of emerging aneuploid and hyperploid cells during tumorigenesis by the immune system (115). These mechanisms fall under the ‘immunosurveillance’ system, and work in tandem with cell-intrinsic checkpoints to promote cell death in response to DNA damage (115). Specifically, prior work in cell lines and mouse models has shown that aneuploid and hyperploid cells trigger signals that recruit cytotoxic T cells and natural killer cells, resulting in immunogenic cell death (116, 117). It has been hypothesized that aneuploid cancer cells which survive ‘immunoselection’ may contain mechanisms which allow them to become unrecognizable to immune cells, either by reducing their expression of tumor and adjuvant signals, or by sending out other immunosuppressive signals (115). Once evolved, an established aneuploid phenotype suppresses immune infiltration and immune-mediated destruction (115), as evidenced by the association between aneuploidy and immune evasion markers observed across multiple cancer types, including melanoma (22).

Understanding what causes this shift in the relationship between aneuploid cells and immune cells may have implications for predicting response to ICI treatment and identify new therapeutic opportunities. The study by Tripathi et al. (118) used an experimental mouse model of tumor aneuploidy to test the hypothesis that the down-regulation of major-histocompatibility class I (MHC-I) machinery (part of the antigen presentation pathway, or APP), which is critical for effector T cells to target and attack tumors, is responsible for aneuploidy-induced immunosuppression. Indeed, their results support the idea that aneuploid tumors that have survived immunoselection evolve from initially activating pro-inflammatory signaling, to ultimately suppressing this signaling, at least partially *via* the epigenetic silencing of APP genes (118). Future studies utilizing animal models of tumor aneuploidy to analyze tumor biopsies at different stages of disease will be key to elucidate the specific mechanisms by which aneuploid melanoma cells are able to proliferate and evade the immune system in the primary and metastatic setting. As new studies make evident that distinct metastatic sites– e.g. lung versus liver - and mutational profiles mechanistically implicated in aneuploidy (26, 27, 29) – *BRAF* V600E versus V600K, as well as 9p21 loss, where *CDKN2A/B* reside - are associated with differences in ICI treatment response in melanoma (99, 119, 120), future efforts on the understanding of the tumor-immune interaction need to account for organ-specific immune cell composition and mutational background of the tumors at high resolution, as evidenced by a recent study based on single-cell sequencing in uveal melanoma (121). In non-cutaneous and acral melanoma, while the lower mutational load may play a role in the poorer response to ICI therapies, the association with aneuploidy load remains to be assessed.

### Mechanisms that lead to aneuploidy and tetraploidy

It is unclear the extent to which defects in different components of the cell cycle cause aneuploidy and hyperploidy in melanoma. Recent work in matched primary melanomas and precursor lesions by Shain *et al*. has shown bi-allelic inactivation of *CDKN2A* to be a common feature in the transition to invasive melanoma, coinciding with an increase in the extent of aneuploidy (46). Large melanoma cohorts present a ploidy distribution that is bimodal, with a diploid peak and a hyper-triploid peak, with approximately one third of melanomas in the latter category (18, 122). *In silico* assessments estimate that melanomas with increased ploidy have undergone at least one round of WGD throughout tumor evolution (18, 42, 122), suggesting defects that lead to tetraploidization including mitotic slippage, cell-cell fusion and cytokinesis failure are common. A defective decatenation checkpoint, frequently observed in melanomas, was shown to lead to failed cytokinesis (83), suggesting this could be a prevalent mechanism by which a hyper-triploid state is achieved.

Studies implicating *BRAF* V600E mutation with SAC dysregulation, loss of *CDKN2* with centrosome amplification and *KIT* K642E mutation with replication stress prompt the characterization of aneuploidy and tetraploidy on large cutaneous (4, 123) and mucosal melanoma cohorts (40) segregated by mutational background, including the *TP53* tumor suppressor. TP53 is key to controlling the emergence of tetraploidy (124). Still, *TP53* mutation varies across mutational backgrounds of cutaneous melanomas, with *BRAF*-mutant, *NRAS*-mutant, *NF1*-mutants and triple wild-type melanomas presenting with 12%, 20%, 29% and 7% of *TP53* mutations, respectively. In one pan-cancer study (122), while WGD was significantly more present in *TP53*-mutant tumors across cancer types, only 25% of melanoma *TP53*-mutant tumors had a WGD event, a similar rate to that observed in the entire melanoma cohort. Future work with mouse models that incorporate these mutational backgrounds and weakened checkpoint signaling (125–127) could shed light on the emergence of aneuploidy and tetraploidy in melanoma.

Other defects that have been shown to lead to aneuploidy in other cancers may contribute to aneuploidy across melanoma subtypes. Entosis (128) – the internalization of another cell of the same or different type - has been shown to generate aneuploid cell lineages by causing cytokinesis disturbances in the host cell in breast tumors (129). In human melanoma, cell-in-cell structures have been directly observed, e.g. in the form of melanoma-specific CD8+ T cells engulfed and digested by metastatic melanoma cells (130). Global methylation loss is a common trait of cancer (131) and has been causally linked to genomic instability and aneuploidy *via* DNA methyltransferase (DNMT) knock-out (132). In melanoma cell lines that constitutively express *PD-L1*, an association between reduced expression of *DNMT3A* and DNA hypomethylation levels was identified (133). The role of these and other mechanisms that can result in aneuploidy deserve further investigation in melanoma.

## Conclusions and perspectives

Significant progress has been made on the characterization of aneuploidy in melanoma due to the development of technologies that enable visualization and quantification of the karyotype. Large cohorts have unveiled common and distinct recurring events across melanoma subtypes, as well as differences in the frequency of aneuploidy, with uveal melanomas distinctively carrying lower levels compared to cutaneous, acral and mucosal melanomas. Underlying drivers of recurring events as well as their potential clinical value as therapeutic targets remain to be elucidated in many cases, and further progress within rare melanoma subtypes - such as those within mucosal melanomas - will require large patient cohorts.

Both recurring aneuploidy events and overall levels of aneuploidy hold promise as diagnostic and prognostic tools in melanoma. Future studies that take into consideration the cellular heterogeneity of benign melanocytic tumors and that measure additional molecular coding and non-coding features in nevi and melanoma should result in improved tools for the identification of malignancy in histologically borderline melanocytic tumors. Understanding of the independent prognostic value of aneuploidy compared to thickness and other clinical-pathological factors relies on future research that systematically assesses aneuploidy across thickness levels, utilizing cohorts with sufficient statistical power and long-term follow-up. As is the case with identification of drivers of disease, the prognostic value of individual aneuploidy events and overall levels of aneuploidy in melanoma subtypes of low prevalence will depend on the generation of large cohorts.

Understanding of the tumor-immune interaction across varying mutational backgrounds and immune contexts in cutaneous and non-cutaneous melanoma subtypes will likely be key to the further elucidation of the role of aneuploidy as a predictive tool in the context of ICI treatment and identification of new candidate targets for patients that fail ICI therapies. To this end, work in animal models as well as cross-sectional and longitudinal designs utilizing single cell technologies will provide unprecedented resolution of the relationship between aneuploid tumors and their microenvironment. Similarly, further knowledge on the relative contribution of distinct mechanistic defects that lead to aneuploidy and tetraploidy in the context of driver mutations should unveil new drug targets of the proliferative and invasive potential of melanomas.

## Author contributions

Conceptualization: IV; data curation: ES, IP, JW, and IV; supervision: GL, RS, and IV; writing of original draft preparation: ES and IV; writing review and editing: ES, JW, IP, GL, RS, and IV. All authors contributed to the article and approved the submitted version.

## Funding

RS and GL are supported by National Health and Medical Research Council of Australia (NHMRC) Practitioner Fellowships, and their research is supported by an NHMRC Program grant. JW is supported by a NHMRC investigator grant. GL is supported by the University of Sydney Medical Foundation.

## Acknowledgments

Support from colleagues at Melanoma Institute Australia and Royal Prince Alfred Hospital are also gratefully acknowledged.

## Conflict of interest

RS has received fees for professional services from F. Hoffmann-La Roche Ltd, Evaxion, Provectus Biopharmaceuticals Australia, Qbiotics, Novartis, MSD Sharp & Dohme, NeraCare, AMGEN Inc., Bristol-Myers Squibb, Myriad Genetics, GlaxoSmithKline. GL is consultant advisor for Aduro Biotech Inc, Amgen Inc, Array Biopharma Inc, Boehringer Ingelheim International GmbH, Bristol-Myers Squibb, Evaxion Biotech A/S, Hexel AG, Highlight Therapeutics S.L., Merck Sharpe & Dohme, Novartis Pharma AG, OncoSec, Pierre Fabre, QBiotics Group Limited, Regeneron Pharmaceuticals Inc, SkylineDX B.V., Specialized Therapeutics Australia Pty Ltd.

The remaining authors declare that the research was conducted in the absence of any commercial or financial relationships that could be construed as a potential conflict of interest.

## Publisher’s note

All claims expressed in this article are solely those of the authors and do not necessarily represent those of their affiliated organizations, or those of the publisher, the editors and the reviewers. Any product that may be evaluated in this article, or claim that may be made by its manufacturer, is not guaranteed or endorsed by the publisher.
